# DNA barcoding for identification of anuran species in the central region of South America

**DOI:** 10.7717/peerj.10189

**Published:** 2020-10-21

**Authors:** Ricardo Koroiva, Luís Reginaldo Ribeiro Rodrigues, Diego José Santana

**Affiliations:** 1 Departamento de Sistemática e Ecologia, Universidade Federal da Paraíba, João Pessoa, Paraíba, Brazil; 2 Instituto de Ciências da Educação, Universidade Federal do Oeste do Pará, Santarém, Pará, Brazil; 3 Instituto de Biociências, Universidade Federal de Mato Grosso do Sul, Campo Grande, Mato Grosso do Sul, Brazil

**Keywords:** Anura, Frog, Mato Grosso do Sul, DNA Barcode, COI, Brazil

## Abstract

The use of COI barcodes for specimen identification and species discovery has been a useful molecular approach for the study of Anura. Here, we establish a comprehensive amphibian barcode reference database in a central area of South America, in particular for specimens collected in Mato Grosso do Sul state (Brazil), and to evaluate the applicability of the COI gene for species-level identification. Both distance- and tree-based methods were applied for assessing species boundaries and the accuracy of specimen identification was evaluated. A total of 204 mitochondrial COI barcode sequences were evaluated from 22 genera and 59 species (19 newly barcoded species). Our results indicate that morphological and molecular identifications converge for most species, however, some species may present cryptic species due to high intraspecific variation, and there is a high efficiency of specimen identification. Thus, we show that COI sequencing can be used to identify anuran species present in this region.

## Introduction

Anurans (Amphibia: Anura), commonly known as frogs and toads, are an extremely endangered group, with 30% of their species threatened (Vitt & Caldwell, 2014). This number is still considered underestimated given recent descriptions and the lack of studies evaluating and monitoring populations and/or determining the status of at least one-third of the known amphibian species (Bickford et al., 2007; Keever et al., 2009; IUCN, 2017; Vacher et al., 2020). The cryptic characters of many species often makes the correct identification of anuran species very difficult (e.g., Funk, Caminer & Ron, 2012), which has promoted the use of molecular approaches to discovery of new species and identification by non-specialists.

In Brazil, the largest country in South America, 1,136 species of the order Anura are known, of which approximately 70% are endemic (Segalla et al., 2019; AmphibiaWeb, 2020). This country contains a wide diversity of biomes from large forest areas such as the Amazon rainforest to open grasslands like the Pampas. Among its biomes, two were classified as biodiversity hotspots in the seminal article by Myers and collaborators: Atlantic forest and Cerrado (Myers et al., 2000). The Atlantic Forest is among the top five biomes labelled biodiversity hotspots, and is the most devastated biome in the country (Rezende et al., 2018). The Cerrado, the second largest biome in Brazil and the richest savannah in the world, constitutes the most extensive savannah region in South America. After the Atlantic Forest, the Cerrado is the Brazilian biome that has suffered the most changes due to human occupation (Colli, Vieira & Dianese, 2020). In addition to these biomes, other regions of the country are of great conservation importance for global species diversity, such as the Pantanal, the world’s richest tropical wetland area that is recognized by UNESCO as a World Heritage Site (Calheiros, De Oliveira & Padovani, 2012).

Considering the biological diversity of these environments, Mato Grosso do Sul is a Federative State in the central region of Brazil that consists of Atlantic forest, Cerrado, and Pantanal biomes. Given its privileged geographic positioning, several initiatives have improved the taxonomic knowledge and conservation of flagship species found in the State, e.g., the Hyacinth Macaw Project (Guedes, 2015). Moreover, other initiatives such as the Ecological-Economic Zoning and species inventories have been carried out to obtain new robust data about environmental threats and species lists for different taxa (Neves et al., 2017; Ferreira et al., 2017; Rodrigues et al., 2018). Until 2017, 86 anuran taxa, assigned to species level, were identified in Mato Grosso do Sul state (Souza et al., 2017). Although Souza et al. (2017) reported the presence of these species, some records have recently been questioned. Santos et al. (2019) suggested removing the species *Boana crepitans* (Wied-Neuwied 1924), *Scinax similis* (Cochran, 1952), *Adenomera martinezi* (Bokermann 1956) and *Leptodactylus gracilis* (Duméril & Bibron 1840). Brusquetti et al. (2014), suggested the incorrect identification of *Lepidobatrachus asper* Budgett, 1899 as *Lepidobatrachus laevis* Budgett, 1899 for the state.

In the last decades, the field of molecular systematics experienced remarkable progress that led to the development of standardized DNA sequences used for taxonomy (e.g., Koroiva et al., 2017; Guimarães et al., 2018). Among these genetic tools, we found the mitochondrial gene Cytochrome Oxidase I (mtCOI, Cox 1 or COI), which was first suggested by Hebert and colleagues, as a basis for creating a global species identification system, especially for animals (Hebert et al., 2003; Hebert, Ratnasingham & de Waard, 2003). Such system was proposed as an efficient tool in the face of the growing need for quick and easy methods to identify species and assist in the discovery of new taxonomic units, considering that only a fraction of biodiversity has been described and named (Mora et al., 2011).

This system called “DNA Barcode” or “DNA Barcoding” has gained a lot of attention from the scientific community. Since the article suggesting a standard threshold value of 1% in BOLD system (Ratnasingham & Hebert, 2007a), hundreds of papers testing DNA Barcode have been published in prestigious scientific journals, demonstrating the efficiency of this tool for biological identification (e.g Schindel & Miller, 2005; Park et al., 2011; Koroiva et al., 2017; Koroiva et al., 2018). In Latin America, the use of DNA Barcoding technique in anurans has been expanded in the last decade. Previous evaluations of the effective use of DNA Barcoding in the specimens identification suggest an accuracy above 75%, especially for anuran species present in the Amazon and the Atlantic Forest (Estupiñán et al., 2016; Lyra, Haddad & De Azeredo-Espin, 2017). Wide-scale DNA barcode surveys of anurans have also been performed in Colombia and Panama with confirmation of its ability for molecular identification (see Paz & Crawford, 2012; Guarnizo et al., 2015). However, in the main biomes of the central region of South America (e.g., Cerrado), molecular information on anurans is still scarce, limiting regional genetic data and the application of new molecular technologies (e.g., metabarcoding).

In regard to the restrictions and difficulties of morphological identification of anurans, molecular tools present a promising approach to solve this impediment in Neotropical species. In this study, we present a comprehensive DNA barcode library for the identification of anuran species in the central region of South America, in particular, for the state of Mato Grosso do Sul (Brazil). Although political borders have no biological basis, biodiversity knowledge produced in a state can direct contribute to the design and monitoring of public policies (Fernandes et al., 2017) and help the planning and management of resources (Pelayo-Villamil et al., 2018). Thus, our objectives were (i) to establish DNA barcode libraries for the Anura fauna of Mato Grosso do Sul state, (ii) to make a comparison between molecular (distance-based and tree-based) methods of species delimitation and traditional taxonomy, and (iii) to evaluate the accuracy of DNA barcoding in identifying specimens.

## Materials & Methods

### Ethics statement

This study was approved by the Brazilian Institute for Wildlife and Environment/Ministry of Environment (SISBIO license number 45889-1 and 49080-1). Protocols for collection and research were approved by the Animal Use Ethics Committee of the Federal University of Mato Grosso do Sul (CEUA/UFMS-1.1096/2019).

### Data collection

A total of 104 anuran specimens of 48 species were collected from 13 municipalities in the state of Mato Grosso do Sul (from now on “MS”). Morphological identification of all specimens was done with the help of experts on anuran taxonomy and by consulting the Coleção Zoológica da Universidade Federal de Mato Grosso do Sul (ZUFMS-AMP). The collected specimens were killed using 5% lidocaine chlorohydrate, fixed with 10% formalin and then transferred to permanent storage in 70% ethanol. We also collected tissue samples and stored them in 100% ethanol before specimen fixation in formalin. For classification, we followed Frost (2020). Voucher specimens were deposited into the collections of the Coleção Zoológica da Universidade Federal de Mato Grosso do Sul, Brazil (ZUFMS-AMP/UFMS).

**Figure 1:**
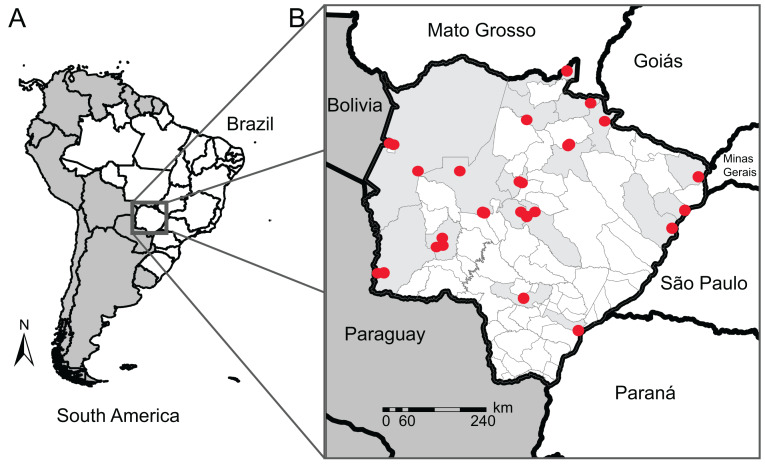
Map of our sampling locations in Mato Grosso do Sul state. (A) Map of South America (dark gray) highlighting the geopolitical division of Brazil (white); (B) municipality division of Mato Grosso do Sul (MS) with the locality of the sampling sites (red dots) and their municipalities (light gray). Collection localities and other specimen information are available in Table S1.

To improve the genetic database, we used sequences from neighboring states (São Paulo, Mato Grosso, Goiás, Paraná, and Minas Gerais) and countries (Bolivia and Paraguay) for species that we did not have sequences for in MS or for those with sequences from one single individual (singletons). COI data from traceable sequences available on GenBank and BOLD System repositories were also included in our reference library. These sequences belong to 22 anuran specimens of 11 species from 7 municipalities in MS sequenced by other authors, totaling 126 anuran specimens of 49 species from 17 municipalities (Fig. 1), and 74 anuran specimens of 17 species from neighboring Brazilian States or neighboring countries of MS. In this count, we sequenced four specimens from two species (*Dendropsophus melanargyreus* and *Osteocephalus taurinus*) collected in Mato Grosso state because these occur in MS state but we did not have access to tissue samples. All sequences were verified to represent the same region of COI gene and checked for indels and stop codons.

### Amplifying and sequencing

The total DNA from the samples was extracted using the Blood & Tissue DNA Mini Kit (Ludwig Biotec, Alvorada, Brazil) from a small piece of toe, muscle or liver tissue preserved in ethanol according to standard DNA barcoding methods for anurans. 658 bp were amplified from the 5′ region of the COI gene using the primers described in Lyra, Haddad & De Azeredo-Espin (2017):

T3-AnF1 –‘5- ATT AAC CCT CAC TAA AGA CHA AYC AYA AAG AYA TYG G-3′

T7-AnR1 –‘5- AAT ACG ACT CAC TAT AGC CRA ARA ATC ARA ADA RRT GTT G-3′

PCR conditions for amplification consisted of 1 × buffer (Colorless GoTaq^®^ Flexi Buffer; Promega Corp., Madison, WI), 0.2 mM dNTP mix, 0.2 µM of each primer, 2mM MgCl2, 1U Taq polymerase (GoTaq^®^ G2 hot start polymerase, Promega Corp., Madison, WI) and 2µl of template DNA, in a total reaction volume of 25 µl. The PCR cycling program was run as follows: initial denaturation step with 3 min at 95 °C, 35 cycles of denaturation for 20 s at 95 °C, annealing for 20 s at 50 °C and extension for 1 min at 60 °C, and final extension for 5 min at 60 °C (see Lyra, Haddad & De Azeredo-Espin, 2017). PCR products were purified with Ethanol/Sodium Acetate and directionally sequenced in ABI 3130 Genetic Analyzer (Applied Biosystems). COI sequencing was performed using T3 (5′- ATT AAC CCT CAC TAA AG - 3′) and T7 (5′- AAT ACG ACT CAC TAT AG - 3′) primers. The sequence data was uploaded to GenBank (accession numbers MT583090 to MT583197).

### Data analysis

We used GENEIOUS v 9.0.5 (https://www.geneious.com) to check the sequence quality of both strands through comparisons with their respective chromatograms, and to assemble and edit if necessary. Furthermore, we aligned sequences for each gene loci using Muscle v3.8.425 (Edgar, 2004) (module implemented in GENEIOUS v 9.0.5) with the default setting. To increase robustness in the homology statement and elevate matrix occupancy, long sequences were truncated to cover only the “Folmer” region of the COI gene. This is the most used region for DNA barcoding and covers 658 nt of the 5′-end of the gene (Koroiva & Kvist, 2018).

### Species delimitation

Two methods were used to delimit species: distance-based and tree-based methods. As a distance-based method, we performed ABGD analyzes online (https://bioinfo.mnhn.fr/abi/public/abgd/), using K2P model and, since the program is optimized for COI gene, the default values of Pmin = 0.001 and Pmax = 0.10, steps = 20, and Nb bins = 20 were used. We also used a value of relative gap width (X), 0.75 to increase the sensitivity of the analysis (Puillandre et al., 2012). For this analysis, we used the K2P model because it is used in other barcoding studies (e.g., Hendrich et al., 2015; Hawlitschek et al., 2016). ABGD resulted in a stable genetic group count with a range of prior intraspecific values (*P* = 0.0234–0.0483) and results of these grouping are presented. For the latter analysis, the best-fit nucleotide substitution model was selected by jModeltest 2.1.7. (Darriba et al., 2012). GTR+I+G was the best fit model available.

For tree-based methods, we used the Poisson Tree Process (PTP and mPTP) as implemented in the PTP web servers (http://species.h-its.org/ and https://mptp.h-its.org) using default settings and removing outgroup sequences (Zhang et al., 2013; Kapli et al., 2017). We constructed a tree with RAxML (v 8.2.12) (Stamatakis, 2014) using GTR GAMMA I model and 1,000 bootstrap replicates. In addition, the computer program BEAST 1.8.4 and packages (Drummond & Rambaut, 2007) were used to infer an ultrametric tree (lognormal relaxed clock, GTR + Γ + G evolutionary model, ucld.mean parameter set to 1 and constant population size coalescent tree prior). Two independent runs of 1.3 10^8^ generations were conducted remotely at CIPRES v 3.3. portal (Miller, Pfeiffer & Schwartz, 2010). Convergence was verified with Tracer v 1.6 and a maximum credibility tree was drawn using TreeAnotator 1.8.4, discarding the first 10% as burn-in. The GMYC analysis (Fujisawa & Barraclough, 2013) was run online (http://species.h-its.org/gmyc/), employing a single threshold method on standard parameters. Finally, a maximum likelihood (ML) tree was created to provide a graphic representation of the divergence pattern between species. ML tree was inferred using PhyML 3.0 (Guindon et al., 2010) website (http://www.atgc-montpellier.fr/phyml/) with 1,000 bootstrap replicates and GTR + I + G model. In all tree-based analysis, three species were used as outgroup (see Table S1).

### Specimen identification

The success of identification was evaluated using the “nearest-neighbor” criterion (also known as “Best match” (BM)), “Best Close Match” (BCM) and “BOLD Identification Criterion” (BIC) analysis using the SPecies IDentity and Evolution (SPIDER v. 1.3) (Brown et al., 2012) in the R program (R Development Core Team, 2020). An in-depth description of each analysis can be found in Talaga et al. (2017) and Lavinia et al. (2017).

To evaluate the ability to identify species using DNA Barcoding gene, we applied the genetic distance threshold values of (i) 1%, as used in the BOLD system (Ratnasingham & Hebert, 2007b); (ii) function “threshVal” in SPIDER (Brown et al., 2012), (iii) 6%, as used and suggested by Lyra, Haddad & De Azeredo-Espin (2017); (iv) 8%, as used and suggested by Crawford, Lips & Bermingham (2010) (from now on “Crawford et al.”); (v) function “localMinima” in SPIDER (Brown et al., 2012); (vi) 10%, as used and suggested by Vences et al. (2005). Singletons were not used as queries. All analyses were performed using K2P model.

## Results

### Sampling and final data set

A total of 204 mitochondrial COI barcode sequences were obtained from 22 genera and 59 species (see Table S1). The data herein represents the first published DNA barcodes for 19 taxa (32%). Four species that were found and sequenced in this research were not in the species list for MS used as a parameter: *Pseudis platensis, Ameerega berohoka, Proceratphrys dibernardoi*, and *Pristimantis dundeei*. All sequences analyzed were greater than 416 bp. The average base pairs of the sequences was 624 bp and the median was 638 bp. Nine singletons were registered for the database: *Rhinella scitula, Physalaemus marmoratus, Leptodactylus furnarius, Leptodactylys syphax, Trachycephaylus typhonius, Pseudopaludicola falcipes, Pseudopaludicola mystacalis, Scinax acuminatus* and *Boana caingua*.

### Species delimitation

**Figure 2:**
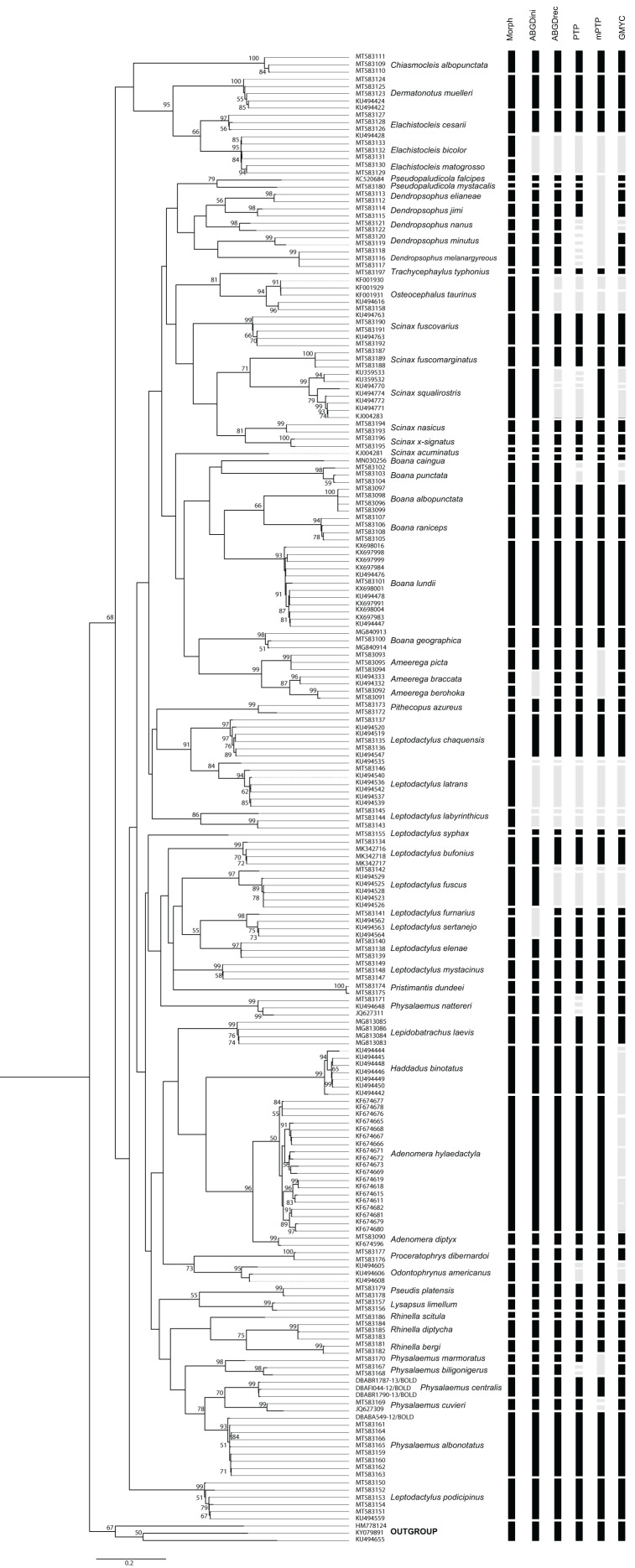
PhyML tree of Anura species based on COI sequences obtained mainly from Mato Grosso do Sul State specimens. The numbers above the internal nodes correspond to bootstrap values of only those higher than 50% are indicated. Black bars represent each species delimited by the following methods: Morph (Morphology), ABGDini (automatic barcoding gap discovery: initial partition), ABGDrec (automatic barcoding gap discovery: recursive partition), PTP (Poisson tree process), mPTP (multi-rate Poisson tree processes), and GMYC (General Mixed Yule Coalescent). Gray bars indicate divergence results from morphological identification.

Species delimitation analyzes provided varying numbers of possible species considering 59 species defined by morphological delimitation: 58 for ABGD-initial partition, 64 for ABGD-recursive partition, 77 for PTP, 53 for mPTP and 72 for GMYC (Fig. 2). Thus, the number of possible anuran species observed ranged from 58 to 77. The results of the species delimitation analysis revealed six conflicts with at least three methodologies disagreeing with the morphological delimitation.

### Specimen identification

Using SPIDER (Table 1), we obtained 195 correct identifications and no incorrect identifications for “Best Match” approach. A value of 2.64% was proposed by “localMinima” function and the value that minimized the cumulative identification errors (“threshVal” approach) was 8.2%. For BCM approach, correct identification varied between 75.39% (147 correct at threshold value of 1%) and 98.46% (192 correct at threshold proposed by the “threshVal” approach, Crawford et al. and Vences et al.; 8.2%, 8.6% and 10%, respectively). For “BOLD identification criteria” analysis, the correct identification varied between 75.39% (147 correct at threshold value of 1%) and 95.38% (186 correct at threshold proposed by the “threshVal” approach and Crawford et al.; 8.2% and 8.6%, respectively).

**Table 1:** Summary of the results from the sequence-based identification simulations. A species name was determined following three criteria: Best Match (BM), Best Close Match (BCM) and BOLD Identification Criterion (BIC). Six threshold values (1.00%, 2.64%, 6.00%, 8.20%, 8.60% and 10.00%) were used for the BCM and BIC approaches. The total number of identifications within each category (values in parenthesis) and the percentage they represent are based on 195 queries (singletons were not considered).

Reference	Threshold value	Criterion	Correct	Incorrect	Ambiguous	No identification
“Nearest-neighbor”	–	BM	100.00% (195)	0.% (0)		
BOLD’s threshold	1.00%	BCM	75.38% (147)			24.62% (48)
		BIC	75.39% (147)			24.62% (48)
“localMinima”	2.64%	BCM	88.21% (172)			11.79% (23)
		BIC	85.13% (166)		3.08%(6)	11.79%(23)
Lyra, Haddad & De Azeredo-Espin (2017)	6.00%	BCM	96.41% (188)			3.59% (7)
		BIC	93.33% (182)		3.08% (6)	3.59% (7)
“threshVal”	8.20%	BCM	98.46% (192)			1.54% (3)
		BIC	95.38% (186)		3.08% (6)	1.54% (3)
Crawford, Lips & Bermingham (2010)	8.60%	BCM	98.46% (192)			1.54% (3)
		BIC	95.38% (186)		3.08% (6)	1.54% (3)
Vences et al. (2005)	10.00%	BCM	98.46% (192)			1.54% (3)
		BIC	93.33% (182)		5.13%(10)	1.54%(3)

## Discussion

This library of DNA sequences represents a major advance for DNA barcodes to identify anurans from central South America. As advocated by Carstens et al. (2013), the application of multiple approaches should increase the reliability in the species identification, although it is necessary to recognize the limitations inherent in the choice of threshold values. Considering three or more methods of delimitation, six taxonomic inconsistencies (10.16%) had better evidence. The presence of complexes of closely related species and problems of taxonomic knowledge gaps may be associated with these results. Below, we discuss the most likely hypothesis for each taxon with disagreement.

*Scinax squalirostris* specimens were clustered into two main groups. Considering that the evaluated sequences are not from MS, other studies have already suggested that this species might contain cryptic species. Magalhães de (2012) suggests the presence of up to three species with divisions in the southern regions of South America, Atlantic forest and Brazil’s central area, which may explain its variation.

In *Leptodactylus*, specifically for *Leptodactylus fuscus*, the *L. fuscus* species group is the most diverse and widely distributed of the genus, with 30 currently recognized species (Sá et al., 2014). Camargo, De Sá & Heyer (2006) suggested the presence of three cryptic species in *L. fuscus*, which highlights this position. Despite our molecular results, as advocated by Struck et al. (2018), studies of cryptic species should be carried out using a multidimensional and interdisciplinary approach, requiring an integrated investigation of characters that can be used to differentiate species. In a recently accepted manuscript Magalhães et al. (in press), *Leptodactylus latrans* presents four cryptic species, which corroborates the variation found in the sequences obtained from GenBank. For *L. labyrinthicus*, Silva et al. (2017) demonstrate that there are limitations to morphological identification, and also suggest the existence of possible cryptic species.

Finally, in a phylogenetic analysis of the genus *Osteocephalus*, Jungfer et al. (2013) recognize five candidate cryptic species within *O. taurinus*. One of the candidate species occurs in the eastern region of the state of Mato Grosso, Brazil, and must occur in sympatry with *O. taurinus* sensu stricto, which explains the high genetic variability of this species. Furthermore, there was one case of merged species: *Elachistoceleis bicolor* and *E. matogrosso*. *Elachistoceleis* is a genus with very similar morphology (Almeida de et al., 2016), and few studies have molecularly evaluated its taxonomy (e.g., De Sá et al., 2012). Considering the conserved morphology, geographic proximity of these species, and that the main difference between them is based on coloration traits (Caramaschi, 2010), we believe that these reasons can cause bias in identification.

Regarding specimen identification, as registered by Raupach et al. (2015) in marine crustaceans from the North Sea and adjacent regions, the BM approach provided the greatest number of correct identifications. This statement should be considered with caution, considering that incomplete databases with few specimens per species may not represent accurate intraspecific genetic variation. Using the distance limits (BCM and BIC) we were able to highlight more cases of high intraspecific divergence (“No identification”) than interspecific divergence, indicating the potential for new species in the state and nearby regions. Recent descriptions of new species found in MS have indicated that this hypothesis appears to be correct (e.g., Pansonato et al., 2016). The threshold value of 8.6% suggested by Crawford, Lips & Bermingham (2010) who worked in a more restricted geographical area (highlands of central Panama), is close to that found for the ‘threshvall’ approach (8.2%). These values presented the best evaluations in both BCM and BIC. Regardless, such proximity to threshold values must be emphasized, as it cannot be considered a standard value to other regions.

Finally, the use of a single value for DNA barcoding have been frequently questioned because several evolutionary factors must be considered in a community such as distinct mutational rates, which is related to their biologic aspects (e.g., size, growth rate, generation time and length), and the condition of the genetic difference in recently separated species (see Meyer & Paulay, 2005; Austerlitz et al., 2009). However, the use of databases and thresholds on regional scales has been encouraged considering that these approaches can increase identification accuracy (Bergsten et al., 2012). Here, we must highlight that the specimen identification capability is high (75.38–98.46%), even for congeneric species, such was also demonstrated by previous studies (e.g., Lyra, Haddad & De Azeredo-Espin, 2017), supporting the use of DNA Barcoding for species-level identification of Neotropical anurans.

## Conclusions

Establishing an Anuran DNA barcode library for central region of South America, especially in Mato Grosso do Sul State, was a landmark to improve the taxonomy and biodiversity conservation in Brazil. Even without all the species, our results demonstrate the ability to identify most of them with the COI gene. Also, this database is an important baseline study for the application of mass sequencing tools for biomonitoring; a current trend in this area. Our results indicate that morphological and molecular identifications are converging for most species, however, some molecular evidence suggests the presence of cryptic species within certain species.

##  Supplemental Information

10.7717/peerj.10189/supp-1 Supplemental Information 1 Sequences also available at GenBank

10.7717/peerj.10189/supp-2 Supplemental Information 2 List of specimens used in this study Shown in yellow are the estimate coordinates of the locality or city center, considering that these records did not have this information.

## Additional Information and Declarations

### Field Study Permissions

The following information was supplied relating to field study approvals (i.e., approving body and any reference numbers): This study was conducted with the appropriate permission from the Brazilian Institute for Wildlife and Environment/Ministry of Environment (SISBIO/IBAMA license number 45889-1 and 49080-1).

### DNA Deposition

The following information was supplied regarding the deposition of DNA sequences: The sequence data are available at GenBank: MT583090 to MT583197.

### Data Availability

The following information was supplied regarding data availability: The list of specimens used in this study is available in a Supplemental File.
